# From Trade Ideas to Professional Ideals

**Published:** 1902-02

**Authors:** 


### Editorial.

### FROM TRADE IDEAS TO PROFESSIONAL IDEALS.

The claim of the International Dental Journal to the attention and support of all practitioners follows from the fact that the International is the only dental magazine in the country conducted by members of the profession solely for the aid and advancement of the profession. Every other magazine is controlled and published by a dental bureau, advertising and having for sale an extensive line of dental appliances. Against these magazines no criticism is directed or intended. Certainly a manufacturing company which combines in its magazine advertisements of its wares with communications and editorials of use to the profession does so with perfect propriety. But, on the other hand, it is no less evident that every learned profession should conduct its own magazine for purely professional purposes. No taint or suspicion of trade ends should attach to this magazine; no possibility should be allowed of professional opinion being warped towards the advancement of sales, and the advertising section should be open on equal terms to the large dealer and to the small one with perhaps half a dozen valuable and unique specialities.

Time was, within the memory of many practitioners now living, when trade ways and ideas dominated the dental profession; besmirched its character, discredited its followers, and made ridiculous the just claim of dentistry to an honored place among the specialized branches of medicine. But from motives of self-preservation, if for no other, the dental profession began to separate a generation ago from the trade idea. Repute and prestige were thereby gained for all dentists, and an obligation was imposed upon them to make this divorce between the trade and the profession absolute and unqualified, both for their own sakes and for the generation that follows.

The INTERNATIONAL DENTAL JOURNAL, published without capital, dependent for its enlargement and continued success upon subscriptions to its issues, comparing favorably, it is believed, with any magazine now published in the value of its reading matter, communications, and editorials, offers, it can hardly be denied, an opportunity to every practitioner to subscribe, at but little expense to himself and with large benefit to the dental world, to the principle to whose triumph this magazine is dedicated.

